# Suprachiasmatic nucleus-wide estimation of oscillatory temporal dynamics

**DOI:** 10.1371/journal.pcbi.1012855

**Published:** 2025-03-06

**Authors:** Yifan Yao, Scott Pauls, Duncan Foley, Tomoko Yoshikawa, Sato Honma, Ken-Ichi Honma, Ellie McVeigh, Nicolas C. Foley, Rae Silver

**Affiliations:** 1 Department of Psychology, Columbia University, New York, New York, United States of America; 2 Department of Mathematics, Dartmouth College, Hanover, New Hampshire, United States of America; 3 Department of Economics, Emeritus, The New School for Social Research, New York, New York, United States of America; 4 Organization for International Education and Exchange University of Toyama, Toyama, Japan; 5 Research and Education Center for Brain Science, Hokkaido University, Sapporo, Japan; 6 Center for Sleep and Circadian Rhythm Disorders, Sapporo Hanazono Hospital, Sapporo, Japan; 7 Department of Psychiatry, Columbia University Medical Center, New York, New York, United States of America; 8 New York State Psychiatric Institute, New York, New York, United States of America; 9 Department of Neuroscience and Behavior, Barnard College, New York, New York, United States of America; 10 Department of Pathology and Cell Biology, Columbia University Medical School, New York, New York, United States of America; 11 Zuckerman Institute Affiliate, Columbia University, New York, New York, United States of America; University of Cincinnati College of Medicine, UNITED STATES OF AMERICA

## Abstract

The suprachiasmatic nucleus (SCN), locus of a circadian clock, is a small nucleus of approximately 20,000 neurons that oscillate with a period of about 24 hours. While individual neurons produce circadian oscillations even when dispersed in culture, the coherence and robustness of oscillation of the SCN as a whole is dependent on its circuitry. Surprisingly, the individual neurons of the intact SCN do not all oscillate in phase with each other. To understand the oscillatory dynamics across the intact nucleus, we develop a model of the relation of the phase of neurons to their PER2 expression at a particular subjective time (CT1900) using *time series data* from SCN slice preparations. Next, we use the model, which produces a surprisingly good fit in the SCN slice data, to estimate oscillator phase at a single time point (CT1900) in *snapshot* data from PER2 expression measurements in intact, unsliced SCN-wide tissue. To monitor temporal changes in phase in time series data, we use PER2::LUC imaging in an ex vivo SCN slice preparation. To study phase in the intact SCN at a fixed time point we use data generated by PER2 staining and a tissue clearing protocol. Because PER2 expression, as measured in the time series slices and the snapshot intact SCN are not directly comparable, the model estimated from time series slices to the snapshot intact SCN data requires a calibrating constant. The results indicate that our model provides a surprisingly good fit to the SCN slice data and is therefore a meaningful method for estimating phase in the intact SCN snapshot data, permitting the study of virtual interventions such as virtual tissue slicing. We next compare oscillation in circuits in the SCN-wide tissue to those that have been disrupted by virtual slicing using a Kuramoto model to simulate the dynamics. The results support prior evidence that the damage done by coronal slicing has the most disruptive impact on SCN oscillation, while horizontal slicing has the least damage. The results point to the importance of connectivity along the caudal-to-rostral axis and indicate that SCN circuit organization depends on the caudal-to-rostral flow of information. In summary, the construction of this model is a major finding of the paper. Our modeling allows us to perform the previously impossible analysis of oscillatory dynamics in static data in an intact SCN captured at a single time point.

## Author summary

A mammal’s internal circadian clock, critical to its function and survival, is created and maintained by the suprachiasmatic nucleus (SCN). The SCN comprises about 20,000 oscillatory neurons that, due to their connectivity, synchronize to the external cycle of day and night. Understanding the dynamics of the oscillation of the tissue provides evidence of how the SCN encodes information about the environment such as daylength and seasonality.

We created a model that allows for the estimation of the phase of the oscillation across the entire SCN, side-stepping the limitation of existing techniques that calculate phases that require slicing and damaging the tissue. The construction of this model is a major finding of the paper. Using this model to inform a simulation of the dynamics, we find that slicing does indeed change the oscillatory dynamics and that the most common orientation in preparing brain tissue slices (coronal) is the most damaging.

## Introduction

**SCN as the master clock:** Maintaining synchrony with the circadian cycle of its environment is necessary for living organisms’ survival, and virtually all species have developed enabling timekeeping mechanisms. In mammals, the suprachiasmatic nucleus (SCN) serves as a master clock to synchronize physiological activities to the regularly recurring cycling of environmental cues caused by the earth’s rotation, especially the light-dark cycle [1]. The SCN is localized above the optic chiasm in the hypothalamus, comprising roughly 20,000 neurons [2,3]. One characteristic that differentiates the SCN from many peripheral clocks is that the SCN retains an autonomous circadian rhythm even without synchronizing to external inputs [4], a feature that requires intra-SCN synchronization among its neurons. Although autonomous intra-SCN synchronicity is well recognized, the oscillatory dynamics of intra-SCN network are not well understood. Based on the current knowledge, the oscillation within the SCN is not homogeneous across the entire nucleus. Instead, there is the directionality of the wave of activity spreading over the SCN, leading to phase differences at different locales within the nucleus [5–7].

**The cellular clock:** The mechanism of oscillation at the cellular and molecular level is the transcriptional-translational feedback loop (TTFL) involving a series of clock genes and their proteins [8,9]. Briefly, the TTFL refers to the process by which the proteins CLOCK-BMAL1 dimers activate the transcription and translation of *per* and *cry* genes. The accumulation and heterodimerization of PER and CRY proteins interact with CLOCK-BMAL1 dimers, which in turn inhibit transcriptional activity of *per* and *cry* themselves. The further degradation of the existing PER-CRY dimer relieves the repression of their own transcription and initiates a new cycle of activities in the TTFL.

**Method to monitor oscillations in the SCN:** Genetically modified animals are extensively used in assessing the oscillatory activities of the TTFL. For instance, SCN tissue slices harvested from mice expressing the fused protein of PER2 (a subtype of PER) and LUCIFERASE (the enzyme producing light when interacting with luciferin) is used to assess the oscillation of PER2 expression (PER2::LUC) [10–12]. By adding luciferin to the culture medium, the bioluminescence intensity reflects the amount of PER2 allowing for tracking of changes in expression level in real time. This method produces invaluable data for analyzing the time series of the expression level of TTFL components. Work in the PER2-LUC oscillation of the SCN slices shows a spatio-temporal wave across the tissue, with more dorsal SCN neurons phase-advanced relative to the rest of the SCN [13–16]. However, there are limitations in this method as it is based on physically sliced SCN tissue. The slicing procedure itself interrupts intra-network connectivity. Specifically, sagittal slicing disrupts the connection along the medial-lateral axis; coronal slicing disrupts connection between the rostral-caudal axis; horizontal slicing disrupts connection between dorsal ventral-axis.

**Method to acquire whole volume imaging of SCN:** One way to bypass slicing problem Is by studying the intact SCN using volumetric imaging such as light-sheet microscopy combined with brain clearing techniques such as iDISCO [17,18]. Using this method, the expression of PER2 in the intact SCN can be visualized without any physical slicing. This procedure preserves the connectivity of the SCN in all orientations. However, there is also a cost. The imaging is done in fixed tissue and time series data cannot be generated from a single sample, making the dataset as a “snapshot” of a specific time point. While it is empirically possible to create a time series by studying many animals sacrificed at selected circadian times, given the high cost and uncertain reliability of inference across brains from different individuals, this question will be best pursued with the development of new techniques for in vivo observations or 3D cultures.

**Goal of the current study:** Here, we aim to decipher the spatiotemporal organization of the PER2 oscillation in the SCN at the resolution of a single cell. We first construct a model from time series data in cultured SCN tissue slices to predict phase, based on the PER2::LUC intensity. We then apply this model to the snapshot data in the iDISCO cleared sample to describe the phase distribution in an intact SCN. Because PER2 measurements in cultured SCN tissue slices and in iDISCO cleared data are not directly comparable, this step requires us to assume a calibrating constant, as described below. The results of this modeling allows us to analyze oscillatory dynamics in the intact SCN, which previously has not been possible, and is the key finding we report here. We next use snapshot data and associated phase estimation to simulate dynamics on both intact SCN and virtually created slices of SCN tissue sectioned in the horizontal, sagittal, or coronal plane. Simulation results support the hypothesis that sectioning distorts dynamics, with coronal slicing doing the most damage, followed by sagittal, and then horizontal, a second major finding. This observed anisotropy complements earlier results showing directional biases in strength of connectivity across the SCN [14]. These results demonstrate the directionality of an oscillatory wave within the SCN and the risk of tissue slicing method, which emphasizes the need for examining the circadian variations at the whole tissue level.

## Background and orientation to the data

### Time series data

**Fig 1:**
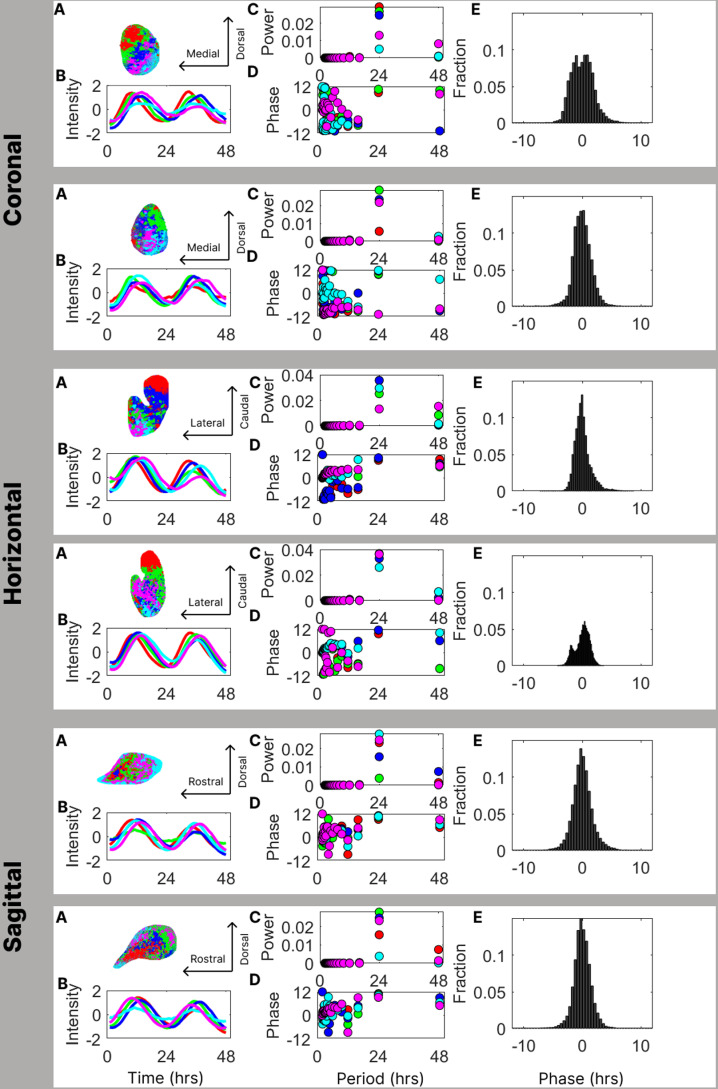
Analysis of time series slice data. Each row reports measurements on one lobe of an SCN, physically sliced and imaged in one of the three orientations – coronal, horizontal, and sagittal. Each SCN was measured hourly, over two days, resulting in a list of 48 measurements at each pixel in the image. **Panel A** in each row the image is rendered in pixels, which are grouped together in 5 color-coded “clusters” as has been done in previous studies [6,19,20] ordered by latency of peak onset. The clustering shows a spatial structure inherent in the dynamics. For panels B, C, and D, the colors indicate the clusters shown in panel A. **Panel B** displays the time series of the average brightness of each cluster, visibly showing a strong circadian component in each series and the order in which the clusters oscillate. **Panel C** shows the Fourier power spectra shown in each row and demonstrates the dominance of the 24-hour period component of the signal as its power is substantially higher than all the other components. Many of the components occlude one another as they are tightly grouped. In **Panel D** the Fourier phase spectra highlight the importance of the 24-hour period component as we see a transition from an incoherent phase spectrum for ultra-circadian period components to a tightly clustered phase profile in the circadian component. In **Panel E** we show the histograms of phases for all the pixels in the tissue - shown to indicate the scope of variation in phase.

In order to link the spatial organization of the SCN to the period and phase of oscillating neurons, we perform time series analysis on the timing and intensity of PER2::LUC bioluminescence expression over several days (Fig 1) on previously published data [14]. By grouping the pixels together in 5 color-coded “clusters”, we see a spatial structure inherent in the dynamics (Panel A) as has been done in previous studies [13,19] with similar results. The time series analysis of the average brightness of each cluster, reveals a strong circadian component in each cluster and shows that the clusters are not in phase, but have a stable order of oscillation (Panel B). To emphasize this, we color the clusters and time series in the order of the peaks of the clusters’ oscillation. Using Fourier analysis on the cluster time series, we can decompose the signals into their oscillator components by period. The power spectra analysis, shown in Panel C, demonstrates the dominance of the 24-hour period component of the signal as its power is substantially higher than all the other components. The Fourier coefficients for shorter “ultra-circadian” periods (higher frequencies) are not exactly zero, since there is some high frequency fluctuation, but they are close to zero. The Fourier coefficients for longer “infra-circadian” periods (lower frequencies) are also not exactly zero, since the time series shows some decay over the experiment. We see this in the Fourier coefficients at the longest measurable period, but these are also very small compared to the dominant circadian signal. Consequently, the Fourier component with a 24-hour period captures almost all the information in the signal.

This feature of the data has several important implications for our analysis. First, the high power at the circadian frequency supports the conclusion that the phase measurements at the circadian frequency are likely to be accurate. Second, we can compress the data greatly from 48 observations for each pixel to 2 statistics, namely the average brightness at that pixel, and the phase of its circadian oscillatory component, with highly conserved signal information -- the median of the total power captured by the circadian component is 62%. Further, previous work [13] shows that filtering out the circadian component results in a non-rhythmic signal. Consequently, knowledge of the average brightness and circadian phase alone allows us to reconstruct a very good approximation of the variation of brightness over time as a cosine function with the specified phase advance or delay. The phase spectral analysis shown in Panel D again highlights the importance of the 24-hour period component as we see a transition from an incoherent phase spectrum for ultra-circadian period components to a tightly clustered phase profile in the circadian component. In fact, in all the SCN shown, phases are so tightly clustered together at the 24-hour period that they overlap and occlude one another. This result reinforces the observations from Panels A and B that the oscillation across the tissue has differences in phases that follow a stable spatial pattern across the tissue. The histograms of phases for all the pixels in the tissue, shown in Panel E, indicate the scope of variation in phase. All the histograms are tightly centered at zero phase and are supported almost entirely in the range of phases between a 5-hour advance and a 5-hour delay. The second horizontal slice (row 4) shows a mild bimodality.

### Snapshot data

Light sheet microcopy and tissue clearing protocols allow us to create three-dimensional images of neurons in SCN tissue distinct from other matter, enabling the creation of a 3D snapshot of PER2 expressing neurons, indexing their location and expression level at the time of the tissue is harvested.

**Fig 2:**
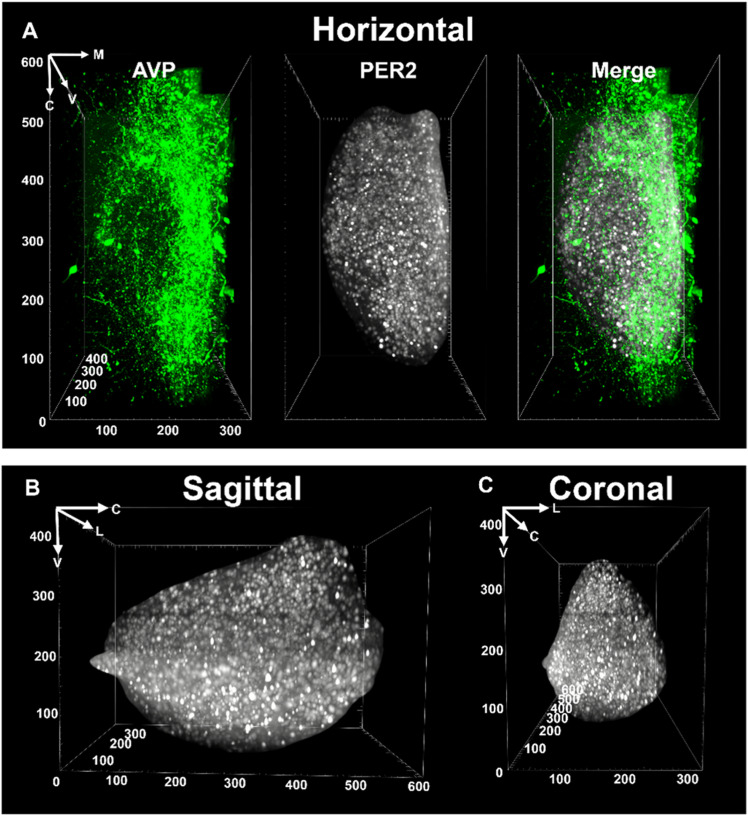
“Snapshot” image of the intact SCN at ZT 1900h in a 3D iDISCO-cleared mouse brain, shown in views from horizontal, sagittal and coronal orientations. Panel **A:** The extent of the SCN is delineated by staining with AVP (left). We show PER2 expression in SCN neurons (middle) and a merged view of AVP and PER2 (right). Panels B and C show the sagittal and coronal views respectively of PER2 expressing SCN neurons. Abbreviations: AVP=vasopressin; C=caudal; L=lateral; M=medial; R=rostral; V=ventral. Axis units=μm.

This snapshot data [17,18] enables us to visualize PER2 expression of individual neuronal oscillators in the intact SCN at a specific point in time (Fig 2). This data overcomes the main limitation of time series data in slices, as it provides information of the state of the intact SCN *in vivo*. But we can only perform the measurement at a single point in time in any given sample - we do not have additional time points with which to perform time series analysis. As a solution, we find that applying our model from time series data in SCN slices to the snapshot data provides an estimate of phase, and consequently the oscillatory dynamics, across the entire, intact SCN.

## Results

### Estimating phase from brightness using time series data

We first ask if PER2::LUC expression levels in *ex vivo* SCN slices given in snapshot data can provide reliable information about phase in the same tissue. To achieve that goal, we develop a method to estimate the phase of neuronal oscillations in cultured SCN tissue using brightness of PER2::LUC expression. Given evidence of anisotropy in strength of connectivity [14] we analyze oscillations in each of the three standard slice orientations - coronal, horizontal, and sagittal shown in Fig 1.

Sinusoidal models of oscillatory signals are determined by four parameters, their frequencies (*ω* ), amplitudes (A), phases (*ϕ*), and additive constants (C):


$$Asin\left(\omega t+\phi \right)+C.$$

Estimating four parameters with data from one point in time should, *a priori,* be difficult if not impossible – as data from one time point provides only one constraint on 4 parameters. Another confounding factor arises due to periodicity – the value at a single time point may indicate the ascending or descending portion of the curve. Time series data, however, has additional structure that constrains the model: across the whole tissue the periods (and hence the frequencies) of the individual neurons are fixed at the circadian period of 24 hours; the constants and amplitudes are constrained; and the phase distribution is tightly clustered around zero (shown in Fig 1E). The latter feature diminishes the ambiguity introduced by periodicity: two phases that would yield the same value at a given time must be twice the distance between either phase and the trough or peak between them. For some time points, particularly at the midpoint between trough and peak, phases would need to be more distant from one another than shown in the results of the time series data phase distributions.

We start by exploring the utility of a linear model – this is the most parsimonious choice and, even if unsuccessful, would yield clues about non-linearities. The linear model we propose linearly relates the difference in phases, Δϕ, of a neurons oscillation to the difference in its PER2::LUC expression, ΔI Formally we have,


ΔI=αΔϕ+c+ϵ, (1)

where *α* is the slope, *c* is an additive constant, and *ϵ* is the residual term.

We use time series data from six SCN – two of each orientation - to build this model. Time series data provide access to phases through the Fourier transform, as described above, and looking at each of the data sets at a single point in time provides an analogue of snapshot data. Integral methods, like the Fourier transform, have a significant advantage over other methods of estimating phase, as they use information from the entire time series. As time series data is typically noisy, phase estimations using integral methods provide better phase estimates due to the denoising associated with integration as well as having less sensitivity to outlier data points. Using the phases from the 24-hour period component of the signal as “ground truth,” we then turned to estimating a linear model relating brightness and phase. We preprocess the data at a single time point by Z-scoring intensity values across all pixels in a given frame, removing the top and bottom 10% intensities, and collect values into 400 equally sized bins (see Methods for details). Because phase is a relative measurement, we relate the *differences in brightness* to *differences in phase* in this linear model.

**Fig 3:**
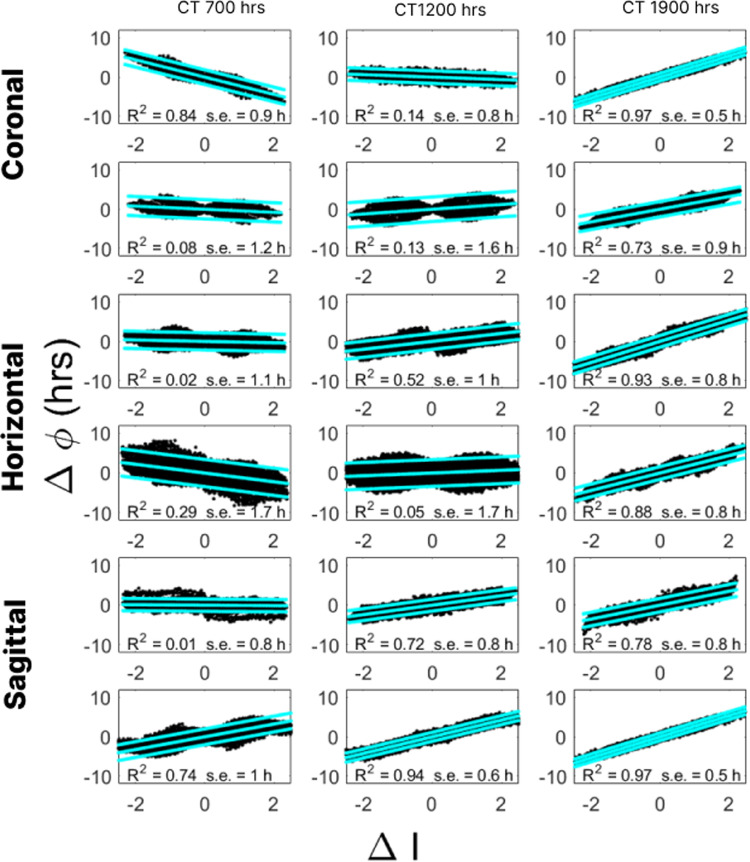
Regression results relating change in intensities to changes in phase in time series data. The scatterplot in each panel shows the pairs $\left(\Delta I_{ij},\Delta \phi_{ij}\right)$ for each pair of pixels $\left(i,j\right)$in the frame, no matter how close or far apart, where $\Delta I_{ij}$ is the difference of the intensities of the pixels and $\Delta \phi_{ij}$ is the difference of the phases as calculated using the Fourier transform. The columns show the results of data taken at **t** = CT 700 hours (left), CT 1200 hours (middle), and CT 1900 hours (right) as measured from the trough of the overall oscillation. The cyan lines show the resulting regression line as well as the envelope of 2 standard deviations. Each panel reports the $R^{2}$ and standard error values for the regression model. In all instances the p-value associated to the regression was less than machine tolerance. We repeat the analysis for the same six sets of time series data as reported in Fig 1, two coronal (top), two horizontal (middle), and two sagittal (bottom) slices.

Upon examining linear fit for each hourly time point starting at the trough of oscillation as hour 0, we were surprised at the remarkable effectiveness of a simple linear fit at CT 1900 hours. For each data set, we found that differences in phases were simply multiples of the differences in brightness – the constant terms, *c*, in the linear fits were all very close to zero, with all 95% confidence intervals including zero with a maximum width of 0.047 and associated p-values below machine tolerance. We constructed linear regression models for each hour in a 24-hour period starting at the trough of the overall oscillation. In Fig 3, we show the linear regression models at hours CT 0700, CT 1200, and CT 1900 timed from the trough of the oscillation (at CT 0000).

We draw three conclusions. First, linear model is particularly effective at CT 1900. The $R^{2}$ values are quite large across our samples (> 0.73), showing that the model captures a large portion of the variance in the data. The p-values for every model were below machine tolerance indicating statistical significance. Further, the standard errors are low (< 1.11 hours), indicating that the models predict phase accurately. Last, the slopes of the regression lines all have the same sign, indicating robustness of the model – if the signs varied, it would show that in some samples a positive difference in intensity is associated with a positive difference in phase while in others, a negative difference in phase. However, while having the same sign the slopes vary in their magnitudes. We address how to calibrate a general model in the next section.

In contrast to the results at CT 1900, the models at CT 1200 are quite poor with low $R^{2}$ values and slopes of different signs. The models at CT 0700 are better than CT 1200 but are not consistent across all the samples.

### Calibrating the model based on time series data

The results at CT1900 give us evidence that support the simplest of linear models,


$$\phi_{i}-\phi_{j}=\alpha \left(m_{i}-m_{j}\right),$$

where the $\left\{\phi_{i}\right\}$ are the estimated phases, the $\left\{m_{i}\right\}$ are the measurements of bioluminescence, and *α* is a parameter. As the $\left\{m_{i}\right\}$ are normalized so that their mean is zero and as phase is a relative measurement, we can use this model to estimate the phase $\phi_{i}$ relative to the global mean phase, which we set to zero and associate with the mean bioluminescence:


$$\phi_{i}-0=\alpha \left(m_{i}-0\right),$$

or


$$\phi_{i}=\alpha m_{i}.$$

The resulting model has a single parameter *α* that determines the appropriate rescaling of the measurements into estimates of phase. The value of this parameter determines the spread of the distribution of estimated phases - if we choose it inappropriately the resulting phase estimates will not be reflective of the dynamics. Estimating the slope *α*, however, is not obvious from the regression models as the slopes for each of the examples in Fig 3, column 3, are different, ranging from 0.33 to 0.70.

To calibrate the model, we use an additional piece of information from the slice time series data -- the level of synchronization of oscillations across the tissue at *CT 1900* hours as measured by the *order parameter* (see Methods). The order parameter is a summary statistic of the phase distribution that quantifies synchronization in the oscillation of the neurons. It is a statistic between zero and one, with zero indicating when the phases of the oscillators are randomly and uniformly scattered, and one, when all the phases are identical, and the oscillators are completely synchronized. For our six samples of time series data, the order parameters are {0.7581, 0.7699, 0.8090, 0.8442, 0.9146, 0.9193} with a mean of 0.8358. To calibrate a snapshot model for a given set of brightness measurements $\left\{m_{i}\right\}$we find *α* by fixing the order parameter of the resulting collection of phase estimates to be 0.84. The choice of *α* has only limited impact on the model – higher or lower values simply raise or lower the spread of the resulting phase distribution. Consequently, the choice of *α* has no impact on appropriately normalized statistics (e.g., skewness, kurtosis) but will have an impact on unnormalized statistics (e.g., mean, standard deviation). Our calibration using the order parameter ensures that the dispersion of estimated phases is similar to those of the time series data.

### Application of model to SCN-wide snapshot data

The model we developed in the previous section allows us to estimate oscillatory phase whenever we have measurements of the PER2 concentration, as time courses of PER2::LUC [12,21,22] are very similar to those of PER2 [23–25]. One source of such information is the snapshot data generated by the iDISCO tissue clearing methodology.

Applying our phase estimation model to snapshot data provides insight into the properties of the oscillatory dynamics of the entire, intact SCN. We estimate phases for data for six SCN from adult animals imaged at *t = CT 1900* hours of their oscillation. Calibration of the six models produce *α* values of $\left\{3.1\times 10^{-4},2.2\times 10^{-4},1.6\times 10^{-4},1.7\times 10^{-4},2.9\times 10^{-4},3.1\times 10^{-4}\right\}$.

**Fig 4:**
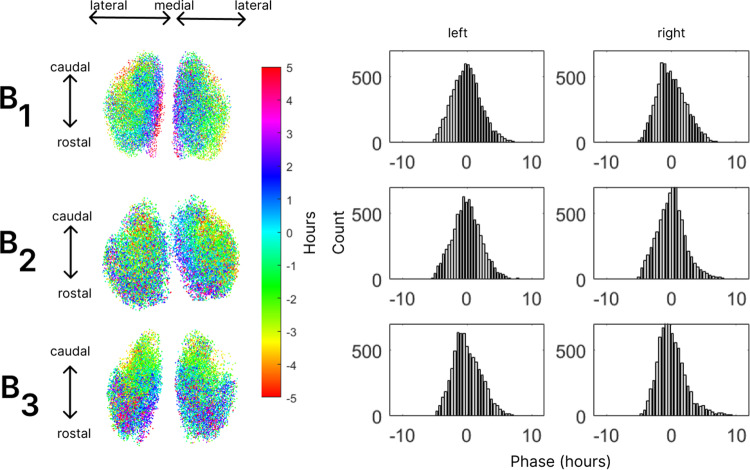
Visualization and properties of phase distributions for snapshot data. The left panel shows the results of estimating phase for snapshot data using the calibrated linear model. We show six SCN, two from each of three brains (B1, B2, and B3) - where the points are located at the center of each of the scanned neurons and color indicates the estimated phase. To the right, the panels show histograms of the estimated phase distributions for the corresponding panel to the left.

Fig 4 shows the phase estimation across each of the SCN (left) and histograms of the associated phase distribution (right). All SCN show heterogeneous phases that progress across the tissue and the histograms are remarkably similar. To support this observation, in Table 1 we report skewness and excess kurtosis of all the phase distributions shown in Fig 3 (note that the means of the distributions are all zero). All estimated phase distributions are skewed right-ward and are leptokurtic. To compare these to the time series data, Fig E in S1 Text shows the skewness and excess kurtosis values shown in Table 1 as well as those for the time series data and for virtual slices (see Methods) of the intact data – all of which, with three exceptions, have positive values of both.

**Table 1:** We report skewness and excess kurtosis of the 6 phase distributions associated to the left and right SCN from three specimens, $B_{1},B_{2},B_{3}$The means of all these distributions is zero.

	$B_{1}$ left	$B_{1}$ right	$B_{2}$ left	$B_{2}$ right	$B_{3}$ left	$B_{3}$ right
skewness	0.3486	0.3983	0.3041	0.5871	0.5238	0.9195
kurtosis	0.3416	0.1876	0.5443	1.4919	1.1518	2.7698

The estimation of phase from PER2 data in intact cleared SCN, using the statistical model based on time series from cultured slice data, is the key finding of this research, and the foundation for further investigations we report below.

In our discussion of time series movies, we noted that physically slicing the tissue for imaging necessarily damages the tissue and may impact and distort the dynamics. With current experimental methods, we cannot directly analyze the intact SCN to determine the impact of slicing on the oscillatory dynamics. Our model allows us to estimate dynamics on the whole SCN and to compare intact dynamics to the dynamics on slices in simulation. That is, having established the properties of dynamics in snapshot data, we can now use the model to see the consequence of different interventions on the dynamics across the entire SCN. We use estimated phase and cell location information from the real SCN-wide snapshot measurements as a foundation for simulation of dynamics in virtually sliced tissue. In executing the model, our first application is to analyze the impact of slicing on the dynamics in a simulation.

For simulation, we use the Kuramoto Coupled Oscillator framework - a system of coupled first order ordinary differential equations where we use the estimated phases, from the phase estimation methods in snapshot data discussed above, as initial conditions (see Methods). Our Kuramoto model requires 3 sets of parameters: a connectivity matrix *A*, a coupling strength Kand a set of initial conditions for the oscillations. *A* is a matrix of zeros and ones where the $ij^{th}$ entry indicates that neurons *i* and *j* influence one another’s oscillations if it is one, and that there is no influence if the entry is zero. The coupling constant, *K*, quantifies the strength of the influence of connected neurons on one another - the larger the value of *K*, the more one neuron pulls the phase of another’s oscillation towards its own.

The precise details of SCN neural connectivity are not completely known (see [26] for a discussion of neural models). We use a well-established first order model of connectivity that assumes the existence of connectivity between nearby neurons complemented by some portion of randomly chosen long-range connections between distant neurons [27], which is reflective of known attributes of neural connectivity (e.g., [28–30]). To construct the matrix *A*, we first connect any neurons within 20 μm of one another from the cell positions obtained in the iDISCO data. We then introduce more connections at random (not derived from iDISCO data) using a parameter $q\in \left[0,1\right]$, which gives the probability of a connection between any two neurons regardless of distance. For our models, *q* and *K* are not determined but instead we simulate over a grid-range of these parameters, $q\in \left[0,0.01\right],K\in \left(0,2\right].$

Kuramoto models are parsimonious in that they make a minimal set of assumptions yet still yield useful results (see, e.g., [31]). However, simulation results can only be broadly interpreted, as the dynamics do not exactly match observed dynamics in time series data due to both the simplicity of the model as well as the assumptions about connectivity. More complex models (e.g., [32,33] and their generalizations) produce more realistic dynamics but come at a much higher computational cost, preventing simulation across the entire SCN. Consequently, to compare dynamics on intact and sliced tissue, we use simulation data on both.

#### Virtual slicing and anisotropy by orientation.

Before presenting the results of our simulations, we return to the snapshot data generated from the iDISCO method to learn what we might expect the results to be from the virtual slicing and simulation. Our goal in this analysis is to see if there are differences in the number of connections when we look in different directions – in other words, if there is heterogeneity in the spatial distribution of neurons. If there are, we would conjecture that virtual slices that “cut” across such a direction would be more damaging than others to the dynamics as more connections would be cut.

We first consider the spatial distribution of the nearest neighbor connections (i.e., the connections given from the data in Awhen q=0). If we denote the centers of the neurons in their three-dimensional spatial coordinates as $\left\{\left(x_{i},y_{i},z_{i}\right)\right\}$, we can consider the collection of directions of the connections,


$$v_{ij}=\frac{\left(x_{i}-x_{j},y_{i}-y_{j}z_{i}-z_{j}\right)}{\left|\left(x_{i}-x_{j},y_{i}-y_{j}z_{i}-z_{j}\right)\right|},$$

for all pairs of neurons i and j. We can represent these vectors in spherical coordinates by finding $\phi_{ij}$ and $\theta_{ij}$ so that


$$v_{ij}=\left(cos\left(\theta_{ij}\right)sin\left(\phi_{ij}\right),sin\left(\theta_{ij}\right)sin\left(\phi_{ij}\right),cos\left(\phi_{ij}\right)\right).$$

**Fig 5:**
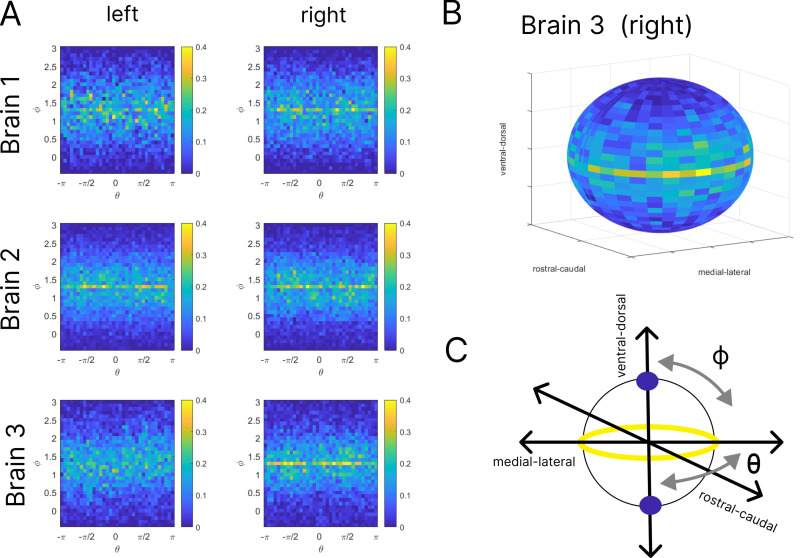
Spatial anisotropy of neural connectivity. The leftmost panels (A) show two-dimensional histograms of the directions of nearest-neighbor connections between neurons in the left and right SCN of 3 brains labeled brain 1,2, and 3. The directions are encoded in spherical coordinates, $\left(\theta ,\phi \right)$. Colors indicate the fraction of all directions that point in a particular direction with yellow shades higher and bluer shades lower. All SCN show the same general result, a concentration of directions around $\phi =\frac{\pi }{2}$ corresponding to directions in the plane spanned by the medial-lateral and rostral-caudal axes. On the upper right **(B)**, we see a mapping of Brain 3 right SCN onto the sphere to aid in interpreting the histograms. On the lower right **(C)**, we show a schematic version of the collective results with the yellow circle representing the concentration band in the histograms and the blue circles indicating the sparsest portions.

If the neurons in the SCN were randomly distributed in every direction, we would expect the $\left\{v_{ij}\right\}$ (and consequently the $\left\{\theta_{ij}\right\}$ and the $\left\{\phi_{ij}\right\}$) to also be randomly distributed. We find that there are preferred directions, which we illustrate in Fig 5. In the left-hand panels of that figure, we visualize the two-dimensional histograms of the distribution of the data derived directions. The rows show the left and right SCN from three brains for a total of 6 SCN. To construct the histograms, we bin the distribution ${\left\{\left(\theta_{ij},\phi_{ij}\right)\right\}}_{i,j}$ into a 35x35 grid with the color representing the count for that bin, i.e., the height of the histogram bar, as a percentage of the total number of neurons, with bluer shades lower and yellower shades higher (see the Fig A in S1 Text for other visualizations). If these distributions were associated with random placement of neurons, the histograms would be almost uniform in color. The concentration of brighter and yellower colors around $\phi =\frac{\pi }{2}$ indicate preferred directions for all 6 SCN, which corresponds to the equator of the sphere (shown in yellow to the lower right of the figure).

The equator lies in the plane spanned by the rostral-caudal and medial-lateral axes in the SCN, indicating a heterogeneity in the distribution of directions of connections. This observation suggests the possibility that slices that impact this plane are likely to more greatly disrupt the resulting dynamics as they sever more connections than slices in other planes.

#### Kuramoto simulation results.

Our analysis of the distribution of directions of potential connections in the SCN gives us hints as to what we might find in simulation – supporting the conjecture that slicing across the horizontal plane will perturb the dynamics more than preserving the horizontal plane. In performing simulations of dynamics over a range of parameters, we evaluate whether slices that cut across horizontal planes impact the dynamics more than those that don’t. Our simulations have two parameters, qand *K*, which determine the connection topology and the extent to which connected neurons influence one another as described above.

For a fixed $\left(q,K\right)$we perform four simulations over a 24-hour period. First, we simulate the intact SCN structure to represent undamaged tissue and dynamics. We then create virtual slices by restricting our model to the neurons within a 100 μm slab of neurons in each of the three standard orientations (see Methods for more details). In these virtual samples, coronal slicing removes the most neurons (averaging 71% of all neurons), sagittal slicing less so (50%), and horizontal slicing the least of all (20%). Restricting the matrix *A* to the remaining neurons, we simulate dynamics on each virtual slice. Comparing the phase at the end of the simulation of the intact (snapshot) SCN to those of the virtual slices provides a measure of the impact of the slicing, which we summarize with a *deviation statistic* given by the mean of the absolute differences between the two sets of phases (see Methods).

**Fig 6:**
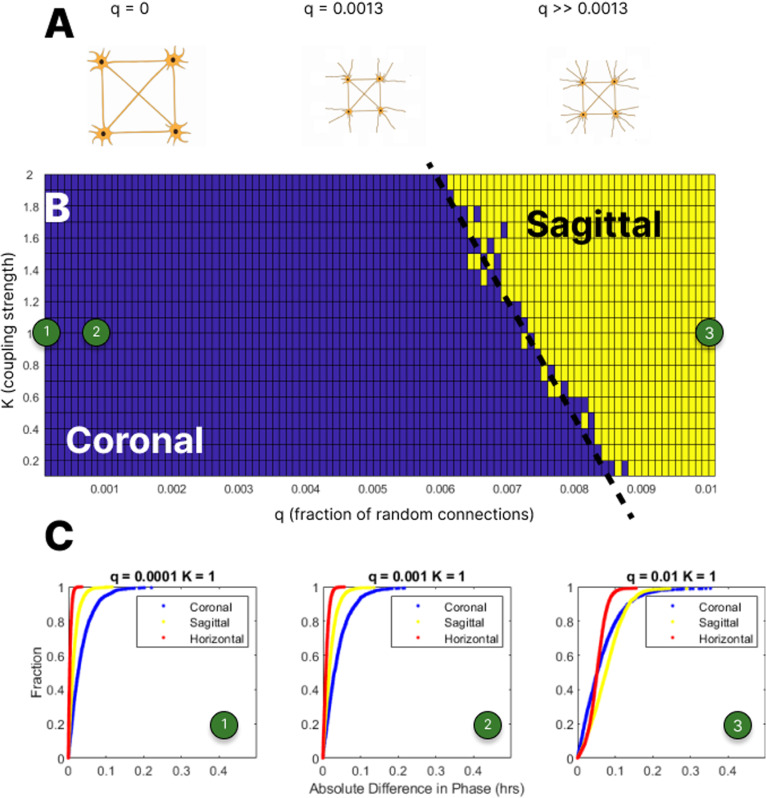
Simulation of dynamical damage from slicing. At the top of the figure **(A)**, we show schematic representations of connectivity given by combining nearest neighbor connections and random connections added with probability *q*. Panel B shows the main results of the simulation indicating which orientation of slicing has the most impact on the dynamics across choices of parameters *q* and K. (*K* is the coupling strength, where the larger the value of *K*, the more one neuron pulls the phase of another’s oscillation towards its own. The parameter $q\in \left[0,1\right]$ gives the probability of a connection between any two neurons). Panel C shows the cumulative distributions of the absolute value of the phase difference between simulation dynamics in the virtual slices and those of the intact neurons. The numbers 1, 2, 3 in circles indicate the specific K and q values as shown in B.

Fig 6 shows the results of these simulations. Panel A shows a schematic of neural connectivity we use in the simulation. In all three schemata, nearest neighbors are connected to one another. Further, random connections are added determined by the probability given by *q*, which increases from left to right. Focusing on panel B, the summary image reports the results of the simulation experiments. For each (q,K) pair we determine which of the three orientations (coronal, sagittal, or horizontal) has the highest deviation statistic and color the pixel – blue for coronal, yellow for sagittal, red for horizontal – accordingly. The image is blue near the origin indicating that the virtual coronal slicing does the most damage to the dynamics. As we move diagonally upwards, increasing both *q* and *K*, there is a line of transition to yellow, indicating that the virtual sagittal slicing does the most damage for those parameter values. We note that there are no red pixels in the image as for all choices of parameter values q and K, the change in dynamics due to horizontal slicing is not the largest. This is consistent with the hypothesis noted above that horizontal slicing does the least damage.

These first results are coarse - we report based on results derived from a single statistic, the mean, for the distribution of the absolute differences. As this may hide relevant details of the differences between the impact of the slices we examine three cases in more detail. In Panel C we show cumulative distribution functions (CDFs) for distributions in three cases: $\left(q,K\right)\in \{\left(0.0001,1\right),\left(0.001,1\right),\left(0.01,1\right).$ We picked the values q=0.0001,0.001, and 0.01 because they reflect three different regimes of the connection topology. The first, q=0.0001, has very few random connections compared to the nearest neighbor connections. The second, q=0.001, is approximately where the number of random and nearest neighbor connections are equal, while in the third, q=0.01, there are many more random than nearest neighbor connections. These three regimes are illustrated schematically in Panel A and are identified in Panel B by green circles labeled 1, 2, and 3. Returning to Panel C, for q=0.001 and 0.01, the horizontal slice CDF (red) is left of the sagittal (yellow) which is left of the coronal (blue). Figs B, C, D in S1 Text show visualizations of the complete simulation results. Coronal virtual slices have mean absolute differences about twice as large as those of the sagittal virtual slices which, in turn, are about three times as large as the horizontal virtual slice means. These results persist for $q\in \left[0.0001,0.001],K\in [0.1,2\right].$

Once we cross the dashed transition line, virtual sagittal slices exhibit more damage on average than the others. We see a more complicated picture of the CDF in the rightmost image in (C) when q=0.01 and K=1. On the left-hand side of the CDFs we see that virtual coronal slices have a larger proportion of small damage than the other two orientations. As we move rightward, the horizontal slice’s CDF quickly moves up towards 1 and the sagittal slice’s CDF moves upwards less quickly. These represent interesting qualitative differences in the impact of virtual slicing in different orientations that merit further future scrutiny.

However, smaller values of q are more biologically plausible as the combination of nearest neighbor connections with a relatively small number of random connections. Existing literature provides evidence that proximate neurons in the SCN are more important to synchronization than those connected at longer ranges. Maywood et al [34] show that local signaling is critical to synchronization and is powerful enough to induce it even in animals lacking VIP. Abel et al [35] show that a functional network model of the SCN exhibits small-world characteristics including dense local connectivity paired with sparse long-range connection. Czeisler et al [36] describe soma-soma contact plates that can facilitate stronger communication contributing to synchronization, further supporting the dominance of local connectivity and communication. Taking all of this together, we see that for biologically plausible portions of the parameter space virtual coronal slicing alters dynamics the most, followed by sagittal and then horizontal slicing. Only with high coupling, high randomness or both do these roles change.

## Discussion

The central contribution of this paper is a tunable linear model for estimating oscillatory phase in the SCN for data on PER2 expression at a fixed time point, namely at CT1900 hours. At this time point PER2 levels are falling in the SCN and have reached half-way to their daily trough. Interestingly, in daily oscillation, this is also the time of maximal interaction of CREB binding protein with the repressor protein PER2, at least in liver tissue [8]. Our model is robust and accurate using time series data from coronal, sagittal, and horizontal slices. Interestingly, in our data estimations from sagittal and horizontal data are better than coronal as the linear models explain more of the variance in the data with smaller standard errors in the former.

Our model extends our ability to examine SCN dynamics to intact tissue using data from iDISCO clearing,a goal that has not been addressed in the published literature to the best of our knowledge. Estimated phase distributions for six SCN from three specimens show remarkable similarities in the shape and spatial organization. We interpret this as further evidence for the usefulness of the model as we would not expect SCN dynamics to radically differ in the two lobes of the SCN or across specimens.

We visually observe spatial structure in the phases as we move through the tissue, echoing the complex spatial structures seen in sliced tissue. For example, several studies have used clustering to examine the spatial distributions of phase using different modalities [13,14,19], while others have identified wave-like structures in phase across the tissue [5,6,15,16,37,38]. These consistencies further support the efficacy of the model estimation.

The estimated phase distribution sets the stage for a comparison of dynamics of sliced tissue and that of intact SCN. Preparing SCN tissue by slicing and plating necessarily removes some of the neural architecture of the SCN, so we could reasonably conjecture that slicing would have impact on the dynamics. However, even with substantial lesioning the SCN retains coherent oscillation so long as a portion of the core tissue is intact [39]. Thus, it is also reasonable to hypothesize that while the dynamics may be perturbed, the overall oscillatory system remains functional after slicing. For example, we might expect the spatial phase waves in slices to differ from a phase wave observed in intact tissue.

In our simulation experiments, we perform the virtual analogue of slice preparation. The results support the hypothesis that slicing impacts the oscillatory dynamics and does so differently for different slice orientations. Virtual coronal slices have the most deviance in their dynamics from simulated intact tissue, while sagittal slices have less and horizontal slice lesser still. This effect may be driven by the amount of tissue removed as coronal slicing removes the most while horizontal slicing removes the least. However, these results are consistent with our analysis of the anisotropy of connection by orientation where we conjecture that slicing that removed directions in the horizontal plane, spanned by the rostral-caudal and medial-lateral axes, would have more impact due to denser connectivity in those directions. The anisotropy in the distributions of neurons in different directions is echoed in the differences in dynamics.

These findings are also consistent with other results in the literature that describe anisotropy in the SCN. Yoshikawa et al [14] demonstrate that increasing connection strength between neurons as we move along the caudal-rostral axis is a potential explanatory mechanism for observed structures, phaseoids, in the spatial distribution of phase. Directional differences are also implicated in encoding of photoperiod. Several authors [40–42] find rhythms in the caudal aspect of the SCN leading the rostral aspect when entrained to long days. Similarly, phase waves moving along the caudal-rostral axis have been observed using coronal and horizontal slices [6,43,44].

Taken together, we see an emerging picture of a communication topography in the SCN where different regions and communication along different directions have different importance to the oscillatory functions and potentially play different roles. Directions in the horizontal plane directions are particularly important as cutting them produces the largest changes in the dynamics. Coronal slicing produces the most damage highlighting the primary importance of the caudal-rostral communication. Given the ubiquity of coronal slice data in existing analyses and results, our findings point to the potential need to revisit them.

Our model has potential generalization to other types of oscillatory data so long as there is reasonable control over parameters guiding the oscillation. The most important factors are 1) uniform periods across the oscillators when the SCN is synchronized and 2) a sharp-peaked phase distribution. Given their similarity to PER2 data, we expect a version of this model to apply to oscillation of PER1 and Ca+ in the SCN.

## Materials and methods

### Experimental data

#### Ethics statement.

All animal protocols were performed according to protocols approved by the Institutional Animal Care and Use Committee of the Columbia University in accordance with guidelines set by the National Institutes of Health (Protocol AC-AABH1603).

#### Animals.

The PER2::LUC data from coronal, sagittal, and horizontal slices has been reported in prior experiments [14]. For these studies, tissue was harvested between zeitgeber time (ZT) 0500 and 0900 and immediately placed in culture. Data analysis began at least one day later. Circadian time (CT) 0000 was designated as the trough of PER2::LUC expression. For iDISCO preparations, animals were deeply anesthetized at ZT 1900 with ketamine (100 mg/kg) and xylazine (10 mg/kg), then perfused intracardially with 50 ml 0.9% saline followed by 100 ml 4% paraformaldehyde (PFA) in 0.1 M phosphate buffer (PB, pH 7.3). After post-fixing in 4% PFA overnight, brains were transferred to 0.1 M PB with 0.9% saline (PBS).

#### iDISCO Clearing and immunostaining.

The iDISCO tissue clearing protocol was used to visualize the SCN on both sides of the midline as we previously described [17] with the following modification. PER2 antibody (1:250, rabbit, Millipore, Burlington, MA) was used to assess the state of the cellular clock; AVP antibody (1:1000, guinea pig, BMA biomedicals, Augst, Switzerland) delineated the extent of the SCN). The secondary antibodies were as follows: for PER2, donkey anti-rabbit Cy3 (1:200, Jackson ImmunoResearch); for AVP, donkey anti-guinea pig Cy2 (1:200, Jackson ImmunoResearch, West Grove, PA).

A dilution series was run to examine the sensitivity and reliability of the PER2 antibody and optimize the immunostaining. The concentrations of anti-PER2 tested were 1:125, 1:250, 1:500, and no-primary control, incubated with secondary antibody donkey anti-rabbit Cy2 at 1:200. The best signal-to-noise ratio was at 1:250 for anti-PER2. The dilution series for AVP was done in a previous study [45].

#### Light sheet microscopy.

Cleared and immunostained tissues were imaged with Ultramicroscope II (LaVision BioTec, Bielefeld, Germany) equipped with an Olympus MVX10 zoom body (Olympus), a LaVision BioTec Laser Module, and an Andor Neo sCMOS Camera with a pixel size of 6.5 µm. The lasers and filters were as follows: Cy2-AVP was excited at 488nm and emission acquired at 525±50nm; Cy3-PER2 was excited at 561nm and acquired at 605±50nm. The SCN region was scanned horizontally with a voxel size 0.755 x 0.755 x 1µm (LE-RI x R-C x D-V).

#### Image preprocessing and PER2 detection.

Stacks of light sheet microscopy images were imported into Imaris software (Bitplane AG, Zurich, Switzerland) for image rendering. AVP staining was used to delineate the core and shell regions using Imaris Surface tool. Spot tool in Imaris was used for computer assisted identification of PER2-expressing neurons. The estimated XY diameter was set as 4µm and the estimated Z diameter based on point spread function was set as 6µm. The segmentation of cells was done in Imaris with Different Spot Sizes (region growing) method and local contrast background subtraction. The location in and diameter of each PER2 neuron was inspected and corrected manually if needed. The information on position (X, Y, Z coordinates) and intensity of each PER2 neuron was recorded in a spreadsheet for further analysis.

### Analysis

#### Time series data analysis.

For time series data we extract the amplitude and phase associated with the component of the signal with 24-hour period using the Fourier transform, consistent with methods used in other studies [13,19].

For each pixel, we computed a discrete Fourier transform of the first 48 hours of the time series. This results in each pixel having a complex number, α+iβ, associated to the component of signal with a 24-hour period which allows us to compute the amplitude and phase:


$$A=\sqrt{\alpha^{2}+\beta^{2}},\phi =arctan\left(\beta /\alpha \right).$$

Each phase is given in radians, which we convert to hours: $h=\phi \cdot \frac{24}{2\pi }.$ As phase is a relative statistic – it can only be measured against a baseline – we normalize the phases across the SCN so that a phase of zero corresponds with the mean signal across the SCN at the period of 24 hours. This results in the phase of every pixel being at most 12 hours phase advanced, or 12 hours phase delayed relative to the mean oscillation. The product of this process is a matrix that aligns with the images in the frames of the PER2::LUC movie, where each entry is the phase extracted from the time series associated to the pixel in the coordinate in a frame of the movie. Each lobe of the SCN was analyzed separately, and for visualization purposes one of the two lobes was chosen for each of the horizontal and coronal slices.

#### Phase estimation model using time series data.

Our estimation method relies on applying regression to the collection of differences in intensity values and of differences in phases for slice time series data. Given the noisiness of this data, we preprocess via the following steps:

1. Z-score the intensity values across all the pixels in the frame.
2. We remove the 10% highest and lowest intensities.
3. Sort the remaining intensity values into 400 equally sized bins.
4. Create the differences in intensities, which we label ΔIby taking the pairwise differences in the centers of the intensity bins.
5. For each pair of intensity bins, calculate the expected difference in phases between all of the pixels in one bin and all of the pixels in the other. These give us differences in phases, labeled Δϕfor each pair of bins.

Using this preprocessed data, we apply linear regression to provide a model relationship between ΔI and Δϕ


ΔI=αΔϕ+c+ϵ.

#### Order parameter and synchronization.

In studying oscillatory dynamics, we use the *order parameter* to measure the degree of synchronization of the oscillators. If ${\left\{\phi_{j}\right\}}^{N}{}_{j=1}$ is the collection of phases of N oscillators, we define the order parameter as


$$O({\left\{\phi_{j}\right\}}^{N}{}_{j=1})=\frac{1}{N}\left|\sum_{j=1}^{N}cos\left(\phi_{j}\right)+isin\left(\phi_{j}\right)\right|=\frac{1}{N}\left|\sum_{j=1}^{N}e^{i\phi_{j}}\right|$$

Looking at this geometrically, where $cos\left(\phi_{j}\right)+isin\left(\phi_{j}\right)$ represents a point in the complex plane, the order parameter is the distance of the average of all these points to the origin. Consequently, if *O* is near one, the phases are all very close together while if it is close to zero, the phases are spread over the entire circle.

#### Tuning parameters via synchronization.

To find an appropriate slope for our linear model, we use a second piece of information from the slice time series data - the synchronization at CT 1900. If ${\left\{I_{j}\right\}}^{N}{}_{j=1}$ are the median signal intensity in the iDISCO sample across N neurons, we use a representative order parameter value of 0.8 and tune *α* so that


$$O({\left\{\alpha I_{j}\right\}}^{N}{}_{j=1})=\frac{1}{N}\left|\sum_{j=1}^{N}e^{i\alpha I_{j}}\right|=0.8.$$

#### Snapshot data analysis.

##### Virtual slicing and simulation.

To form a virtual slice from iDISCO data, we take a 100 *μ* m slab centered at the mean value of one of the coordinate axes and restrict to the neurons inside. For a virtual coronal slice we take the mean value of the second coordinate (y) so that the slice extends in the medial-lateral and dorsal-ventral directions. Similarly, we use the mean of the first (x) coordinate for a virtual sagittal slice and the mean of the third coordinate (z) for a virtual horizontal slice.

To simulate dynamics on both the intact SCN and virtual slices, we use the Kuramoto coupled oscillator framework [31,46,47], which is defined by a set of coupled ordinary differential equations:


$${\dot{\theta }}_{i}=\omega_{i}+K\sum_{i=1}^{N}A_{ij}sin\left(\theta_{j}-\theta_{i}\right),$$

Here, $\theta_{i}$ is the value of the oscillation for the $i^{th}$ neuron, $\omega_{i}$ is its intrinsic frequency, *K* is the coupling strength, and$A_{ij}$is the connectivity between oscillators *i* and *j*. We fix the intrinsic frequencies to be identical, associated with a 24-hour period. We first define$A_{ij}$to be 1 if neurons *i* and *j* are within 20 *μ* m of one another, measured using the Euclidian distance between the three-dimensional locations of the neurons, and 0 otherwise. Then we set randomly selected entries of the matrix *A* to one with probability *q.* We examine the dynamics over a range of the two parameters *q* and *K*: $q\in \left[0.0001,0.1],K\in [0,2\right].$

We solve these systems over a 24-hour time period using the Runge-Kutta method using the estimated phases for initial conditions and a time step of 30 seconds.

##### Measuring the impact of virtual slicing

For a virtual slice, we define *S* to be the collection of neurons in the slice and *T* to be the set of time steps by hour $T=\left\{0,1,2,\ldots ,24\right\}$ Letting ${\left\{t_{i}\left(j\right)\right\}}_{j\in S.i\in T}$ be the time series generated for the intact SCN and ${\left\{{\hat{t}}_{i}\left(j\right)\right\}}_{j\in S.i\in T}$ be the time series for the virtual slice for a given *q* and *K*, we define the *deviation statistics* as difference between the two:


$$\frac{1}{\left|S\right|}\underset{j\in S}{\sum }\left|\arg e^{i\left({\hat{t}}_{24}\left(j\right)-t_{24}\left(j\right)\right)}\right|.$$

## Supplementary Information

S1 TextA: Three dimensional visualizations of the histograms in Fig 5. B: We present visualizations of the simulation results with parameters q=0.0001,K=1 which is labeled as “1” in Fig 6B and C.In each column we show the oscillatory phase, *θ*, for a virtual slice over the course of the simulation period (top) and the absolute difference between the oscillatory phases of the virtual slice and the phases of the corresponding neurons in the simulation of the intact SCN. The first column shows results from a virtual coronal slice, the second a virtual sagittal slice, and the third, a virtual horizontal slice. The second-row visualizations show the difference in the impact of the different slice orientation qualitatively: the coronal slice shows more widespread changes in simulated phase (i.e., more yellows pixels) than sagittal slicing, which in turn shows more changes than horizontal slicing. C: We present visualizations of the simulation results with parameters q=0.001,K=1 which is labeled as “2” in Fig 6B and C. Interpretation of the panels is the same as in S2a Fig. D: We present visualizations of the simulation results with parameters q=0.01,K=1 which is labeled as “3” in Fig 6B and C. Interpretation of the panels is the same as in S2a Fig. In this case, the sagittal slicing creates more changes than the coronal, both of which create more changes than the horizontal slicing. E: We report the excess kurtosis and skewness for the time series data presented in Fig 1 (red symbols), the virtual slices used in the simulation reported in Fig 6 (blue symbols), and the estimated phases for snapshot data presented in Fig 3 and Table 1 (yellow symbols). All values are positive with three exceptions: one horizontal slice with slightly negative excess kurtosis, and one coronal and one sagittal slice with slightly negative skewness.(DOCX)

## References

[1] Evans J, Silver R. The Suprachiasmatic Nucleus and the Circadian Timekeeping System of the Body. In: Pfaff DW, Volkow ND, editors. Neuroscience in the 21st Century [Internet]. New York, NY: Springer; 2016 [cited 2024 Jun 24]. p. 1–49. Available from: doi: 10.1007/978-1-4614-6434-1_66-3

[2] Moore R, Speh J, Leak R. Suprachiasmatic nucleus organization. Cell Tissue Res. 2002;309(1):89–98. 12111539 10.1007/s00441-002-0575-2

[3] Abrahamson EE, Moore RY. Suprachiasmatic nucleus in the mouse: retinal innervation, intrinsic organization and efferent projections. Brain Res. 2001;916(1–2):172–91. doi: 10.1016/s0006-8993(01)02890-6 11597605

[4] Welsh DK, Logothetis DE, Meister M, Reppert SM. Individual neurons dissociated from rat suprachiasmatic nucleus express independently phased circadian firing rhythms. Neuron. 1995;14(4):697–706. doi: 10.1016/0896-6273(95)90214-7 7718233

[5] Hong JH, Jeong B, Min CH, Lee KJ. Circadian waves of cytosolic calcium concentration and long-range network connections in rat suprachiasmatic nucleus. Eur J Neurosci. 2012;35(9):1417–25. doi: 10.1111/j.1460-9568.2012.08069.x 22501027

[6] Evans JA, Leise TL, Castanon-Cervantes O, Davidson AJ. Intrinsic regulation of spatiotemporal organization within the suprachiasmatic nucleus. PLoS One. 2011;6(1):e15869. doi: 10.1371/journal.pone.0015869 21249213 PMC3017566

[7] Yan L, Okamura H. Gradients in the circadian expression of Per1 and Per2 genes in the rat suprachiasmatic nucleus. Eur J Neurosci. 2002;15(7):1153–62. doi: 10.1046/j.1460-9568.2002.01955.x 11982626

[8] Koike N, Yoo S, Huang H, Kumar V, Lee C, Kim T, et al. Transcriptional architecture and chromatin landscape of the core circadian clock in mammals. Science. 2012;338(6105):349–54. 22936566 10.1126/science.1226339 PMC3694775

[9] Partch CL, Green CB, Takahashi JS. Molecular architecture of the mammalian circadian clock. Trends in Cell Biology. 2014;24(2):90–9. 23916625 10.1016/j.tcb.2013.07.002 PMC3946763

[10] Yamazaki S, Takahashi JS. Real-time luminescence reporting of circadian gene expression in mammals. Methods Enzymol. 2005;393:288–301. doi: 10.1016/S0076-6879(05)93012-7 15817295 PMC3793321

[11] Geusz ME. Bioluminescence Imaging of Gene Expression in Living Cells and Tissues. In: Periasamy A, editor. Methods in Cellular Imaging [Internet]. New York, NY: Springer; 2001. p. 395–408 [cited 2024 Jun 26]. Available from: doi: 10.1007/978-1-4614-7513-2_23

[12] Yoo S-H, Yamazaki S, Lowrey PL, Shimomura K, Ko CH, Buhr ED, et al. PERIOD2::LUCIFERASE real-time reporting of circadian dynamics reveals persistent circadian oscillations in mouse peripheral tissues. Proc Natl Acad Sci U S A. 2004;101(15):5339–46. doi: 10.1073/pnas.0308709101 14963227 PMC397382

[13] Foley NC, Tong TY, Foley D, Lesauter J, Welsh DK, Silver R. Characterization of orderly spatiotemporal patterns of clock gene activation in mammalian suprachiasmatic nucleus. Eur J Neurosci. 2011;33(10):1851–65. doi: 10.1111/j.1460-9568.2011.07682.x 21488990 PMC3423955

[14] Yoshikawa T, Pauls SD, Foley N, Taub A, LeSauter J, Foley D, et al. Phase Gradients and Anisotropy of the Suprachiasmatic Network: Discovery of Phaseoids. eNeuro. 2021;8(5). 10.1523/ENEURO.0078-21.2021 PMC8431825 34385151

[15] Yan L, Karatsoreos I, LeSauter J, Welsh DK, Kay S, Foley D, et al. Exploring spatiotemporal organization of SCN circuits. In: Cold Spring Harbor symposia on quantitative biology. 2007. p. 527–41. 10.1101/sqb.2007.72.037 PMC3281753 18419312

[16] Yamaguchi S, Isejima H, Matsuo T, Okura R, Yagita K, Kobayashi M, et al. Synchronization of cellular clocks in the suprachiasmatic nucleus. Science. 2003;302(5649):1408–12. doi: 10.1126/science.1089287 14631044

[17] Renier N, Wu Z, Simon DJ, Yang J, Ariel P, Tessier-Lavigne M. iDISCO: a simple, rapid method to immunolabel large tissue samples for volume imaging. Cell. 2014;159(4):896–910. doi: 10.1016/j.cell.2014.10.010 25417164

[18] Renier N, Adams EL, Kirst C, Wu Z, Azevedo R, Kohl J, et al. Mapping of brain activity by automated volume analysis of immediate early genes. Cell. 2016;165(7):1789–802. doi: 10.1016/j.cell.2016.05.007 27238021 PMC4912438

[19] Pauls S, Foley NC, Foley DK, Lesauter J, Hastings MH, Maywood ES, et al. Differential contributions of intra-cellular and inter-cellular mechanisms to the spatial and temporal architecture of the suprachiasmatic nucleus circadian circuitry in wild-type, cryptochrome-null and vasoactive intestinal peptide receptor 2-null mutant. Eur J Neurosci. 2014;40(3). 10.1111/ejn.12631 PMC4159586 24891292

[20] Brancaccio M, Maywood ES, Chesham JE, Loudon ASI, Hastings MH. A Gq-Ca2+ axis controls circuit-level encoding of circadian time in the suprachiasmatic nucleus. Neuron. 2013;78(4):714–28. doi: 10.1016/j.neuron.2013.03.011 23623697 PMC3666084

[21] Mei L, Fan Y, Lv X, Welsh DK, Zhan C, Zhang EE. Long-term in vivo recording of circadian rhythms in brains of freely moving mice. Proc Natl Acad Sci U S A. 2018;115(16):4276–81. doi: 10.1073/pnas.1717735115 29610316 PMC5910830

[22] Curie T, Maret S, Emmenegger Y, Franken P. In vivo imaging of the central and peripheral effects of sleep deprivation and suprachiasmatic nuclei lesion on PERIOD-2 protein in mice. Sleep. 2015;38(9):1381–94. 25581923 10.5665/sleep.4974 PMC4531406

[23] Bae K, Jin X, Maywood ES, Hastings MH, Reppert SM, Weaver DR. Differential functions of mPer1, mPer2, and mPer3 in the SCN circadian clock. Neuron. 2001;30(2):525–36. 11395012 10.1016/s0896-6273(01)00302-6

[24] Field MD, Maywood ES, O’Brien JA, Weaver DR, Reppert SM, Hastings MH. Analysis of clock proteins in mouse SCN demonstrates phylogenetic divergence of the circadian clockwork and resetting mechanisms. Neuron. 2000;25(2):437–47. doi: 10.1016/s0896-6273(00)80906-x 10719897

[25] Sudo M, Sasahara K, Moriya T, Akiyama M, Hamada T, Shibata S. Constant light housing attenuates circadian rhythms of mPer2 mRNA and mPER2 protein expression in the suprachiasmatic nucleus of mice. Neuroscience. 2003;121(2):493–9. doi: 10.1016/s0306-4522(03)00457-3 14522008

[26] Senk J, Kriener B, Djurfeldt M, Voges N, Jiang H-J, Schüttler L, et al. Connectivity concepts in neuronal network modeling. PLoS Comput Biol. 2022;18(9):e1010086. doi: 10.1371/journal.pcbi.1010086 36074778 PMC9455883

[27] Vasalou C, Herzog ED, Henson MA. Small-world network models of intercellular coupling predict enhanced synchronization in the suprachiasmatic nucleus. J Biol Rhythms. 2009;24(3):243–54. doi: 10.1177/0748730409333220 19465701 PMC2819153

[28] Pennartz CM, Bos NP, Jeu MT, Geurtsen AM, Mirmiran M, Sluiter AA, et al. Membrane properties and morphology of vasopressin neurons in slices of rat suprachiasmatic nucleus. J Neurophysiol. 1998;80(5):2710–7. doi: 10.1152/jn.1998.80.5.2710 9819275

[29] Long MA, Jutras MJ, Connors BW, Burwell RD. Electrical synapses coordinate activity in the suprachiasmatic nucleus. Nat Neurosci. 2005;8(1):61–6. doi: 10.1038/nn1361 15580271

[30] Drouyer E, LeSauter J, Hernandez AL, Silver R. Specializations of gastrin-releasing peptide cells of the mouse suprachiasmatic nucleus. J Comp Neurol. 2010;518(8):1249–63. doi: 10.1002/cne.22272 20151358 PMC2880332

[31] Acebrón JA, Bonilla LL, Vicente CJP, Ritort F, Spigler R. The kuramoto model: a simple paradigm for synchronization phenomena. Rev. Mod. Phys. 2005;77(1):137.

[32] Forger D, Peskin C. A detailed predictive model of the mammalian circadian clock. Proc Natl Acad Sci. 2003;100(25):14806–11. 14657377 10.1073/pnas.2036281100 PMC299807

[33] Forger DB, Peskin CS. Stochastic simulation of the mammalian circadian clock. Proc Natl Acad Sci U S A. 2005;102(2):321–4. doi: 10.1073/pnas.0408465102 15626756 PMC544301

[34] Maywood ES, Chesham JE, O’Brien JA, Hastings MH. A diversity of paracrine signals sustains molecular circadian cycling in suprachiasmatic nucleus circuits. Proc Natl Acad Sci. 2011;108(34):14306–11. doi: 10.1073/pnas.1101003108 21788520 PMC3161534

[35] Abel JH, Meeker K, Granados-Fuentes D, St John PC, Wang TJ, Bales BB, et al. Functional network inference of the suprachiasmatic nucleus. Proc Natl Acad Sci U S A. 2016;113(16):4512–7. doi: 10.1073/pnas.1521178113 27044085 PMC4843423

[36] Czeisler MÉ, Shan Y, Schalek R, Berger DR, Suissa-Peleg A, Takahashi JS, et al. Extensive soma-soma plate-like contact sites (ephapses) connect suprachiasmatic nucleus neurons. J Comp Neurol. 2024;532(6):e25624. doi: 10.1002/cne.25624 38896499 PMC11419332

[37] Quintero JE, Kuhlman SJ, McMahon DG. The biological clock nucleus: a multiphasic oscillator network regulated by light. J Neurosci. 2003;23(22):8070–6. doi: 10.1523/JNEUROSCI.23-22-08070.2003 12954869 PMC6740506

[38] Davidson AJ, Castanon-Cervantes O, Leise TL, Molyneux PC, Harrington ME. Visualizing jet lag in the mouse suprachiasmatic nucleus and peripheral circadian timing system. Eur J Neurosci. 2009;29(1):171–80. doi: 10.1111/j.1460-9568.2008.06534.x 19032592

[39] Van den Pol AN, Powley T. A fine-grained anatomical analysis of the role of the rat suprachiasmatic nucleus in circadian rhythms of feeding and drinking. Brain Res. 1979;160(2):307–26. doi: 10.1016/0006-8993(79)90427-x 761068

[40] Yan L, Silver R. Day-length encoding through tonic photic effects in the retinorecipient SCN region. Eur J Neurosci. 2008;28(10):2108–15. doi: 10.1111/j.1460-9568.2008.06493.x 19046391 PMC2739445

[41] Evans JA, Leise TL, Castanon-Cervantes O, Davidson AJ. Dynamic interactions mediated by nonredundant signaling mechanisms couple circadian clock neurons. Neuron. 2013;80(4):973–83. 24267653 10.1016/j.neuron.2013.08.022 PMC3841113

[42] Yoshikawa T, Inagaki N, Takagi S, Kuroda S, Yamasaki M, Watanabe M. Localization of photoperiod responsive circadian oscillators in the mouse suprachiasmatic nucleus. Scientific Reports. 2017;7(1):8210. 28811515 10.1038/s41598-017-08186-5 PMC5557852

[43] Sosniyenko S, Hut RA, Daan S, Sumová A. Influence of photoperiod duration and light-dark transitions on entrainment of Per1 and Per2 gene and protein expression in subdivisions of the mouse suprachiasmatic nucleus. Eur J Neurosci. 2009;30(9):1802–14. doi: 10.1111/j.1460-9568.2009.06945.x 19840112

[44] Naito E, Watanabe T, Tei H, Yoshimura T, Ebihara S. Reorganization of the suprachiasmatic nucleus coding for day length. J Biol Rhythms. 2008;23(2):140–9. 18375863 10.1177/0748730408314572

[45] Yao Y, Taub A, LeSauter J, Silver R. Identification of the suprachiasmatic nucleus venous portal system in the mammalian brain. Nat Commun 2021;12(1):1–9. doi: 10.1038/s41467-021-25712-3 34561434 PMC8463669

[46] Kuramoto Y. Self-entrainment of a population of coupled non-linear oscillators. In: Araki H, editor. International Symposium on Mathematical Problems in Theoretical Physics. Berlin, Heidelberg: Springer; 1975. p. 420–2.

[47] Kuramoto Y. Chemical Oscillations, Waves, and Turbulence [Internet]. In: Haken H, editor. Springer Series in Synergetics; vol. 19. Berlin, Heidelberg: Springer; 1984 [cited 2024 Jun 14]. (). Available from: http://link.springer.com/10.1007/978-3-642-69689-3

