# Supplementary material for: Suprachiasmatic nucleus-wide estimation of oscillatory temporal dynamics
Source: PLoS Comput Biol. 2025 Mar 6;21(3):e1012855. doi: 10.1371/journal.pcbi.1012855 (PMC11918361; doi:10.1371/journal.pcbi.1012855)
Supplement: S1 Text — In each column we show the oscillatory phase, θ, for a virtual slice over the course of the simulation period (top) and the absolute difference between the oscillatory phases of the virtual slice and the phases of the corresponding neurons in the simulation of the intact SCN. The first column shows results from a virtual coronal slice, the second a virtual sagittal slice, and the third, a virtual horizontal slice. The second-row visualizations show the difference in the impact of the different slice orientation qualitatively: the coronal slice shows more widespread changes in simulated phase (i.e., more yellows pixels) than sagittal slicing, which in turn shows more changes than horizontal slicing. C: We present visualizations of the simulation results with parameters q=0.001,K=1 which is labeled as “2” in Fig 6B and C. Interpretation of the panels is the same as in S2a Fig. D: We present visualizations of the simulation results with parameters q=0.01,K=1 which is labeled as “3” in Fig 6B and C. Interpretation of the panels is the same as in S2a Fig. In this case, the sagittal slicing creates more changes than the coronal, both of which create more changes than the horizontal slicing. E: We report the excess kurtosis and skewness for the time series data presented in Fig 1 (red symbols), the virtual slices used in the simulation reported in Fig 6 (blue symbols), and the estimated phases for snapshot data presented in Fig 3 and Table 1 (yellow symbols). All values are positive with three exceptions: one horizontal slice with slightly negative excess kurtosis, and one coronal and one sagittal slice with slightly negative skewness. (DOCX) [file pcbi.1012855.s001.docx]

# Supplemental Information


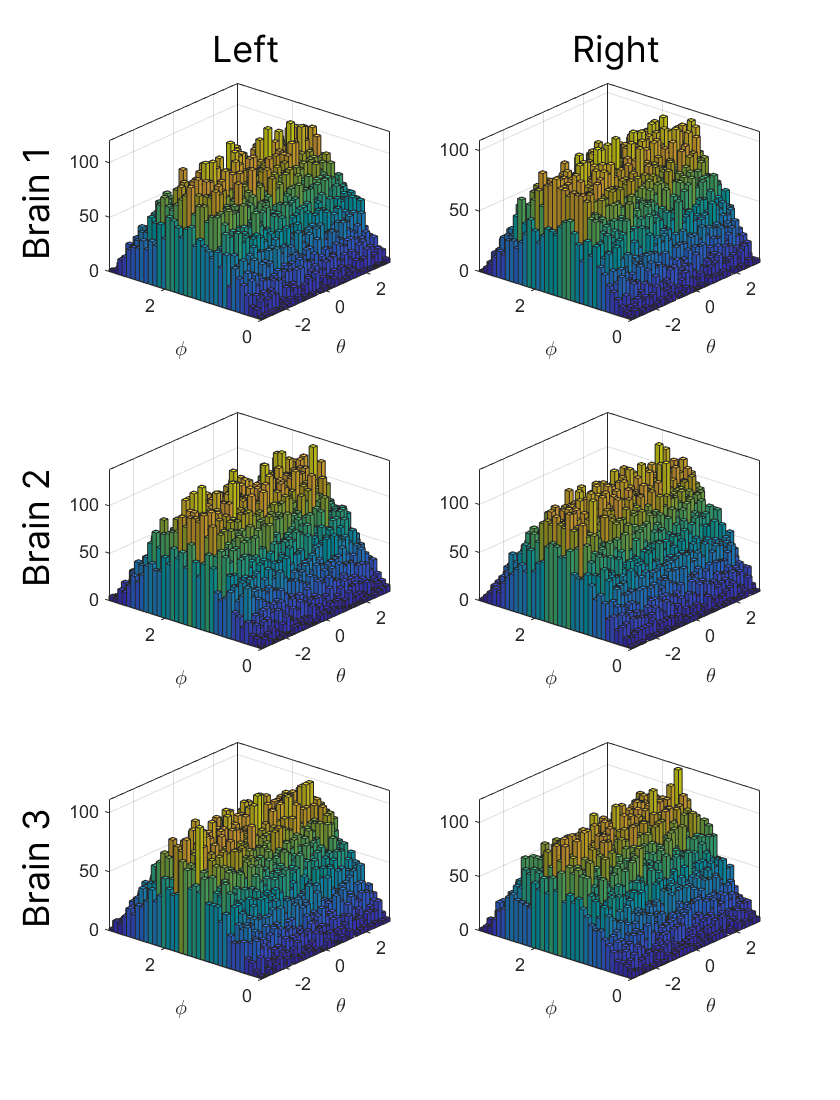


Figure A: Three dimensional visualizations of the histograms in Figure 5.


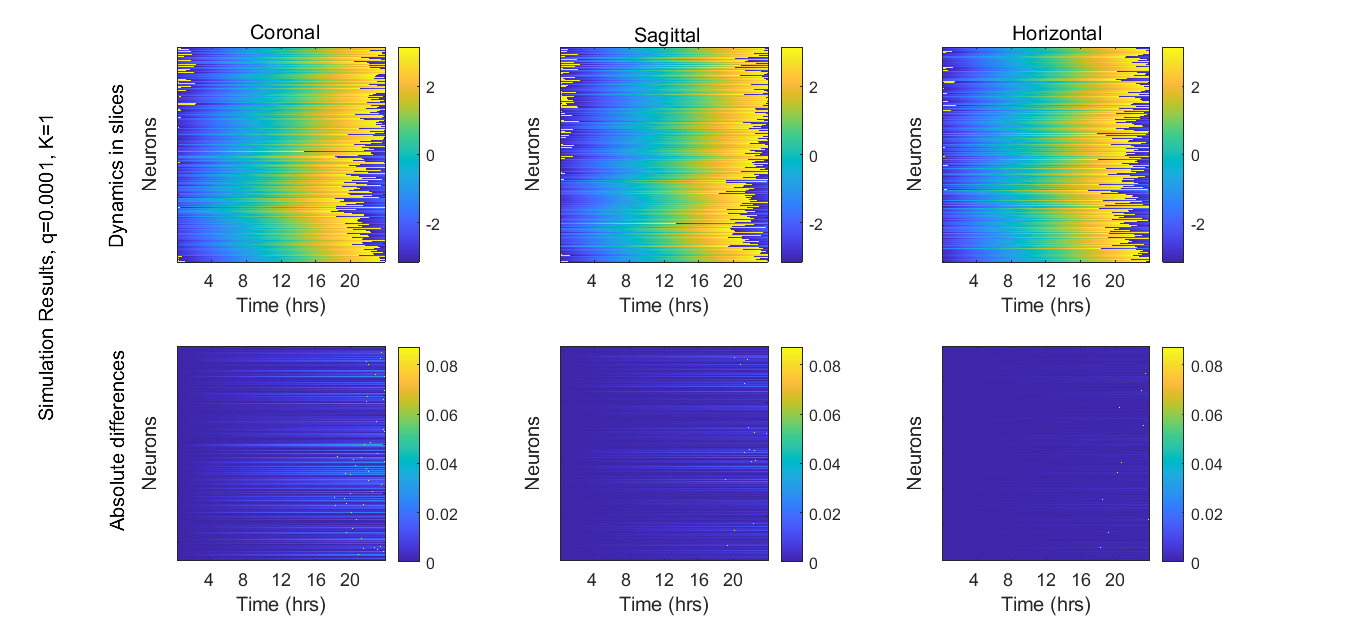


Figure B: We present visualizations of the simulation results with parameters $q=0.0001, K=1$ which is labeled as “1” in Figure 6B and 6C. In each column we show the oscillatory phase, $\theta$, for a virtual slice over the course of the simulation period (top) and the absolute difference between the oscillatory phases of the virtual slice and the phases of the corresponding neurons in the simulation of the intact SCN. The first column shows results from a virtual coronal slice, the second a virtual sagittal slice, and the third, a virtual horizontal slice. The second-row visualizations show the difference in the impact of the different slice orientation qualitatively: the coronal slice shows more widespread changes in simulated phase (i.e. more yellows pixels) than sagittal slicing, which in turn shows more changes than horizontal slicing.


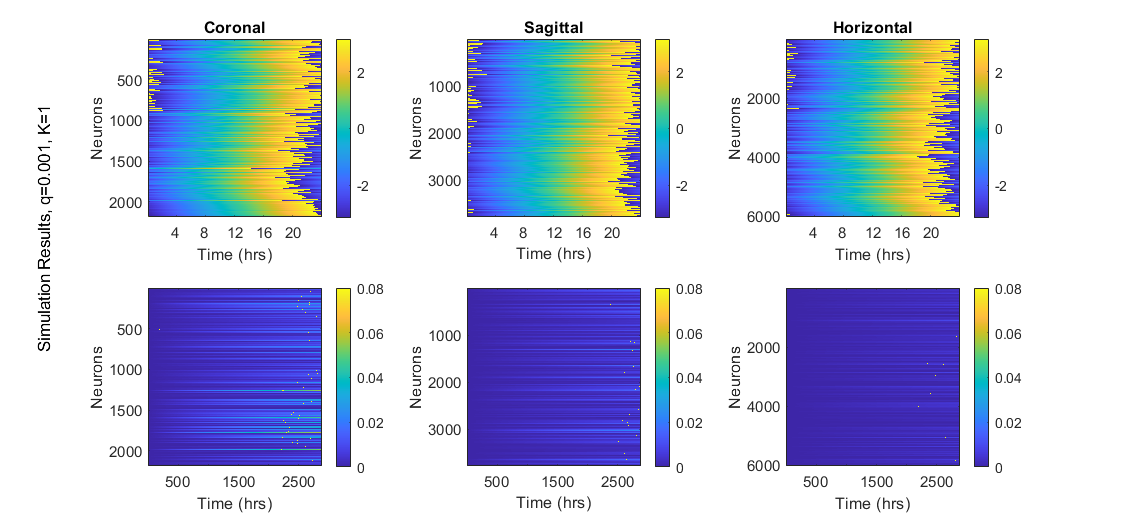


Figure C: We present visualizations of the simulation results with parameters $q=0.001, K=1$ which is labeled as “2” in Figure 6B and 6C. Interpretation of the panels is the same as in Figure Ba.


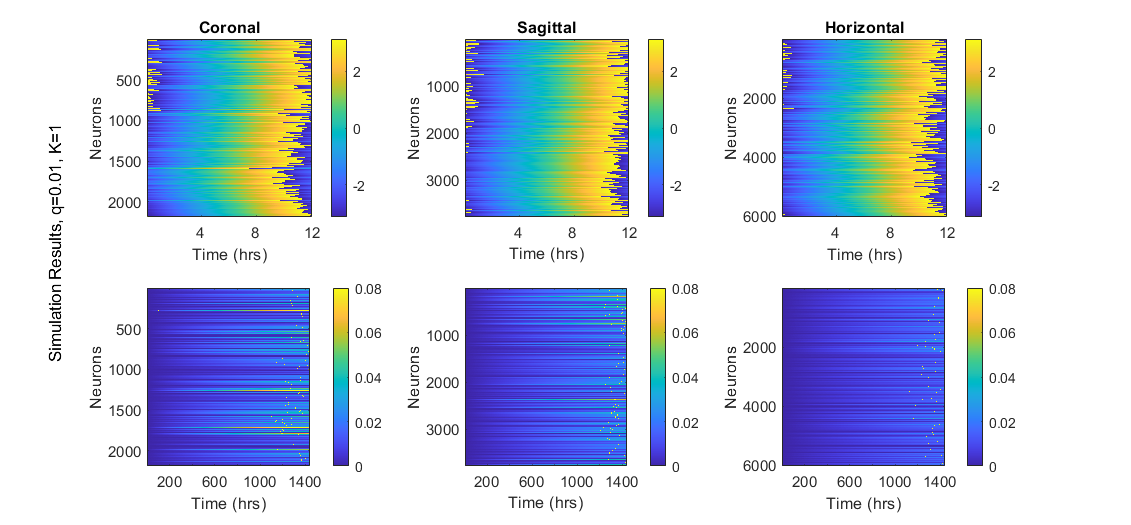


Figure D: We present visualizations of the simulation results with parameters $q=0.01, K=1$ which is labeled as “3” in Figure 6B and 6C. Interpretation of the panels is the same as in Figure Ba. In this case, the sagittal slicing creates more changes than the coronal, both of which create more changes than the horizontal slicing.


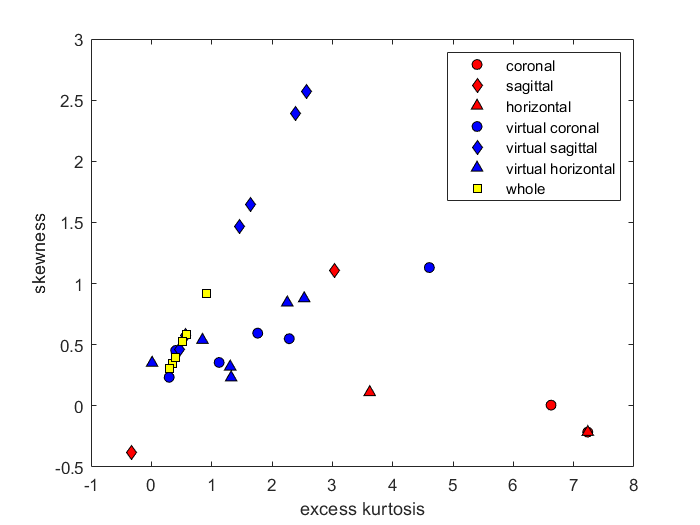


Figure E: We report the excess kurtosis and skewness for the time series data presented in Figure 1 (red symbols), the virtual slices used in the simulation reported in Figure 6 (blue symbols), and the estimated phases for snapshot data presented in Figure 3 and Table 1 (yellow symbols). All values are positive with three exceptions: one horizontal slice with slightly negative excess kurtosis, and one coronal and one sagittal slice with slightly negative skewness.
